# Effectiveness of Workplace Mental Health Programs in Reducing Occupational Burnout: A Systematic Review

**DOI:** 10.7759/cureus.88715

**Published:** 2025-07-25

**Authors:** Abdulaziz Bagasi, Eman K Al Harbi, Salah M Alabbasi, Raneem O Alqaedi, Basmah A Alharbi, Tameem A Alhomaid

**Affiliations:** 1 Family Medicine, Ministry of the National Guard-Health Affairs, King Abdulaziz Medical City, Jeddah, SAU; 2 Family Medicine, Ministry of Health (MOH), Madinah, SAU; 3 Family Medicine, Family Medicine Academy in Madinah, Madinah, SAU; 4 Family Medicine, Ministry of Health Holdings, Jeddah, SAU; 5 Family Medicine, King Fahad Primary Health Care, Madinah Health Cluster, Madinah, SAU; 6 Family Medicine, Ministry of Health Saudi Arabia, Madinah, SAU; 7 Family Medicine, Qassim Health Cluster, Buraydah, SAU

**Keywords:** occupational burnout, organizational interventions, stress management, systematic review, workplace mental health programs

## Abstract

Occupational burnout remains a critical workplace challenge with significant organizational and economic consequences. While workplace mental health programs (WMHPs) are widely implemented, their effectiveness in reducing burnout-related claims requires rigorous evaluation. This systematic review evaluated the effectiveness of WMHPs in reducing burnout-related symptoms.

This systematic review followed Preferred Reporting Items for Systematic Reviews and Meta-Analyses (PRISMA) guidelines to analyze WMHP efficacy. We searched MEDLINE, PubMed, Scopus, and four other databases (2004-2025) for randomized controlled trials (RCTs) and quasi-experimental studies evaluating WMHPs against controls. Two reviewers independently screened records using Covidence, with conflicts resolved by a third reviewer. Study quality was assessed using Cochrane Risk of Bias Tool for RCTs and Joanna Briggs Institute (JBI) Critical Appraisal Checklist for quasi-experimental studies.

This review included 14 studies that met inclusion criteria (nine RCTs, five quasi-experimental; total *n* = 3,572). Studies represented healthcare (*n* = 6), corporate (*n *= 5), and public sector (*n* = 3) settings, with moderate-high heterogeneity in interventions (mindfulness, cognitive behavioral therapy (CBT), organizational restructuring) and outcomes (seven different burnout scales). Participatory organizational interventions reduced burnout for ≥12 months. Digital tools showed short-term benefits, but high attrition (42%), while brief workshops had no sustained effects beyond three months.

Therefore, this review suggests that multi-level WMHPs combining individual and organizational strategies demonstrate the most robust evidence for burnout reduction, though effects vary by implementation quality. This underscores the need for tailored WMHPs that address both individual and structural workplace dynamics to mitigate burnout sustainably.

## Introduction and background

Occupational burnout has emerged as a costly challenge in contemporary workplaces, characterized by emotional exhaustion, cynicism, and reduced professional efficacy [1]. The World Health Organization (WHO) recognizes burnout as an occupational phenomenon resulting from chronic workplace stress that has not been successfully managed [2]. Its impact extends beyond individual well-being, manifesting in diminished productivity, increased absenteeism, higher turnover rates, and substantial financial burdens for organizations, costing employers billions annually in lost productivity and healthcare expenditures [1-3].

The urgency to address occupational burnout has prompted organizations to implement a range of workplace mental health programs (WMHPs). These initiatives, encompassing structured support, mental health screenings, and organizational interventions, aim to foster resilience, improve employee well-being, and ultimately reduce the incidence of burnout symptoms [4,5]. WMHPs are increasingly viewed not only as a moral imperative but as a strategic investment, offering returns such as enhanced productivity, lower absenteeism, reduced presenteeism, and improved employee retention [5,6]. According to the WHO, for every dollar invested in mental health, companies can expect a fourfold return through increased productivity and reduced healthcare costs [7].

Despite the proliferation of such programs, the evidence base regarding their effectiveness (especially in reducing occupational burnout) remains varied. A previous systematic review indicates that while many interventions yield positive effects on burnout or its subcomponents, the magnitude and sustainability of these effects differ across program types and implementation contexts [8]. Person-centered approaches, such as stress management and resilience training, have demonstrated efficacy, particularly when combined with organizational-level interventions that address systemic workplace stressors [8]. However, the long-term impact on occupational burnout symptoms and the comparative effectiveness of different program modalities warrant further investigation.

Recent research underscores the importance of a supportive health and well-being climate (HWC) within organizations. A positive well-being climate, characterized by prevention-focused policies, open communication, and accessible mental health resources, has been shown to reduce both emotional exhaustion among employees [9]. Moreover, supervisor support acts as a critical moderating factor, amplifying the protective effects of a positive workplace climate and further mitigating the risk of burnout and related symptoms [9]. Evidence suggests that the effectiveness of workplace mental health screening programs alone appears to be limited; screening followed by advice or referral shows minimal impact, while screening coupled with facilitated access to treatment offers only modest improvements in mental health outcomes [10]. These findings highlight the need for comprehensive, multi-level interventions that integrate individual and organizational strategies.

Despite the proliferation of WMHPs, critical gaps persist. First, the comparative effectiveness of individual-focused (e.g., mindfulness) versus systemic interventions (e.g., workload adjustments) remains unclear, particularly for long-term outcomes. Second, few studies examine how organizational climate moderates program success [1,9,10]. Finally, cost-effectiveness data are sparse, leaving employers without guidance on optimal resource allocation. This review bridges these gaps by synthesizing evidence across intervention types, contexts, and outcomes. Given the multifaceted nature of burnout and the evolving landscape of workplace mental health interventions, a systematic review is essential to synthesize current evidence on the effectiveness of WMHPs in reducing occupational burnout. Thus, this review aims to critically evaluate the impact of various WMHPs, identify key components associated with successful outcomes, and inform best practices for organizations seeking to safeguard employee well-being and reduce the prevalence of burnout-related symptoms. This review addresses the question: In working adults experiencing workplace stress, how do WMHP, compared to no intervention or standard care, influence burnout reduction, absenteeism, and well-being? By consolidating evidence from recent studies, this study seeks to bridge the gap between intervention research and practical application in diverse occupational settings.

## Review

Methodology

Study Design

This was a systematic review that followed the Preferred Reporting Items for Systematic Reviews and Meta-Analyses (PRISMA) guidelines [11], synthesizing evidence from interventional studies to evaluate the effectiveness of WMHPs in reducing occupational burnout incidences.

Search Strategy 

The research was guided by the question: *In employees experiencing workplace stress, how do WMHPs, compared to no intervention or standard care, influence burnout reduction, absenteeism, and well-being?*

Multiple databases were searched, including MEDLINE, PubMed, Scopus, Web of Science, PsycINFO, Google Scholar, and Cochrane Library, to identify relevant studies. The search combined Medical Subject Headings (MeSH) and keywords related to occupational burnout, workplace stress, mental health programs, and relevant outcomes. For occupational burnout, MeSH terms such as "Burnout, Professional" and keywords like "occupational burnout," "workplace burnout," and "emotional exhaustion" were likely included. For workplace stress, MeSH terms such as "Occupational Stress" and keywords like "job stress," "work-related stress," and "chronic workplace stress" were employed. Regarding mental health programs, MeSH terms like "Mental Health Services," "Employee Assistance Programs," and keywords such as "workplace mental health interventions," "cognitive behavioral therapy (CBT)," "mindfulness training," and "organizational restructuring" were incorporated. For outcomes, MeSH terms, such as "Absenteeism," "Work Engagement," and keywords like "burnout reduction," "stress resilience," "return-to-work rates," and "sickness absence" were included to capture the broader impacts of these interventions. 

These search strings were used: ("Burnout, Professional" OR "occupational burnout" OR "workplace burnout" OR "emotional exhaustion") AND ("Mental Health Services" OR "workplace mental health interventions" OR "cognitive behavioral therapy" OR "mindfulness training") AND ("Absenteeism" OR "burnout reduction" OR "work engagement" OR "sickness absence").

The search was limited to English-language studies published between 2004-2025 to capture 21st-century recent developments in workplace mental health interventions. The last database searches were performed on June 17, 2025.

Study Selection

Inclusion and exclusion criteria: Studies were selected based on predefined criteria. We included peer-reviewed studies focusing on working adults (≥18 years) that evaluated WMHPs such as CBT, mindfulness training, or organizational changes, with measurable outcomes related to burnout reduction. Only interventional studies were included. We excluded studies on acute stress, non-work-related mental health issues, and those lacking quantitative outcome data. Non-peer-reviewed articles, observational studies (whether peer-reviewed or not), and conference abstracts without full texts were also excluded.

Two independent reviewers screened titles/abstracts and full texts based on the aforementioned criteria. Conflicts (*n* = 18 of 369 screened records) were resolved through consensus or consultation with a third reviewer.

Data Extraction and Quality Assessment

Data extraction was performed using a standardized template capturing study design, intervention characteristics, population details, outcome measures, and implementation context. Two independent reviewers conducted the extraction process, with discrepancies resolved through consensus or consultation with a third reviewer. Study quality and risk of selective reporting were assessed using the Cochrane Risk of Bias Tool for RCTs [12] and the JBI critical appraisal tool [13] for non-randomized studies, evaluating factors such as selection bias, performance bias, and outcome measurement.

Data Synthesis and Analysis

The narrative synthesis identified common themes regarding implementation challenges and success factors, such as leadership support and employee engagement. Moreover, subgroup analyses examined variations by intervention type (individual vs. organizational) and delivery mode (digital vs. in-person), and findings were presented in tables. 

Publication bias: Funnel plots were planned to assess this bias, but could not be constructed due to the small number of included studies (*n* = 14) and heterogeneity in outcome measures.

Due to heterogeneity in interventions (e.g., CBT vs. organizational changes) and outcomes (e.g., burnout scales, absenteeism metrics), a meta-analysis was deemed inappropriate. Instead, a thematic narrative synthesis was conducted, including intervention grouping (studies were categorized by type), outcome mapping (effects on burnout, absenteeism, and secondary outcomes), and contextual analysis (moderators, such as leadership involvement and program duration, were identified through iterative comparison).

Results

**Figure 1:**
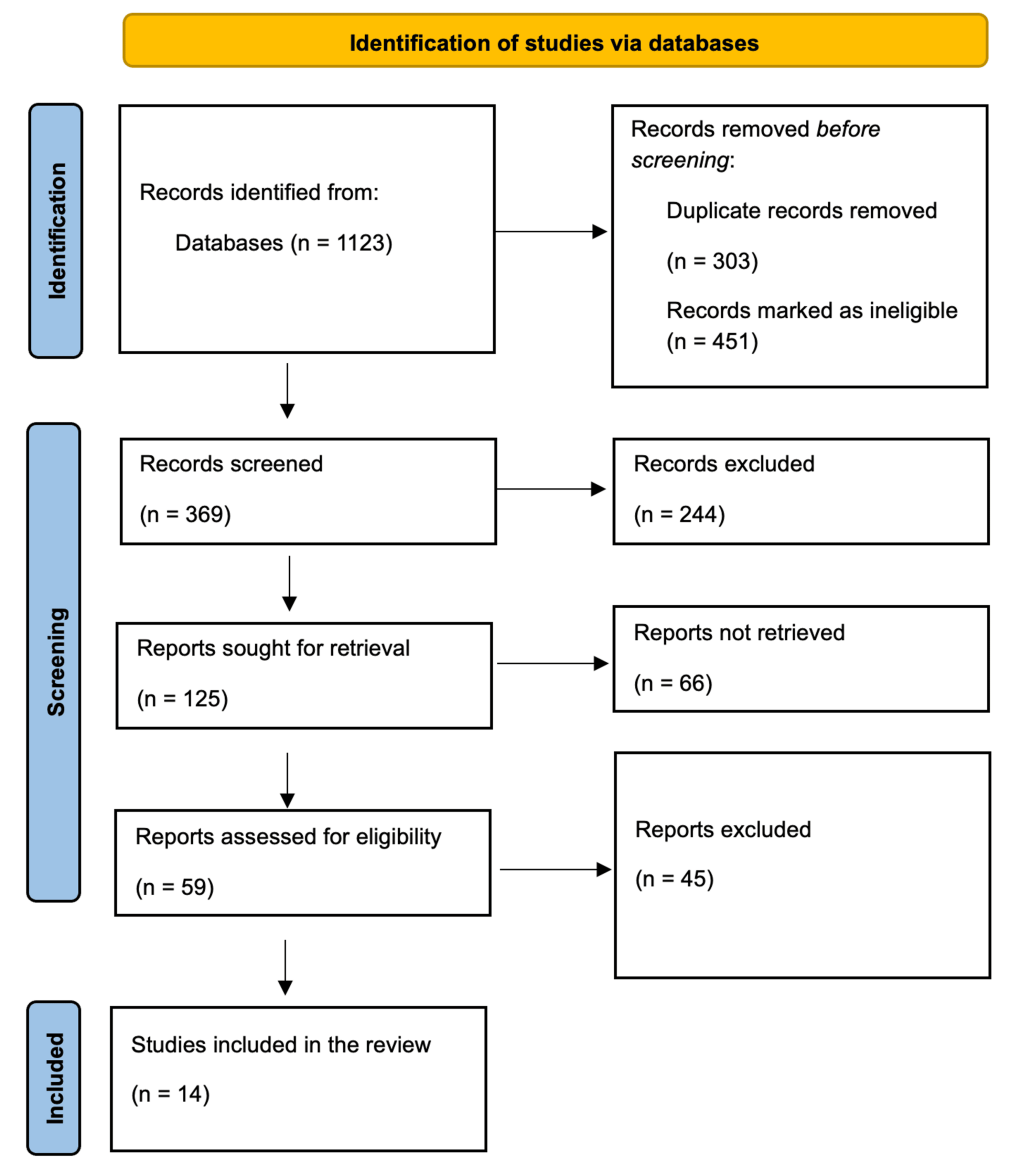
PRISMA flowchart showing the study selection process. PRISMA, Preferred Reporting Items for Systematic Reviews and Meta-Analyses

Initially, we identified 1,123 articles through database searches. After removing 303 duplicate records and excluding 451 ineligible records, 369 records advanced to title and abstract screening. During this phase, 244 articles were excluded for not meeting the inclusion criteria, leaving 125 reports for full-text retrieval. Of these, 66 reports could not be retrieved, resulting in 59 reports undergoing full eligibility assessment. After thorough evaluation, 45 full-text articles were excluded for failing to meet eligibility criteria, resulting in the inclusion of 14 studies for the final systematic review (Figure 1). 

The 14 included studies exhibited considerable diversity in design, population, and interventions. As shown in Table 1, studies ranged from randomized controlled trials (RCTs, *n* = 9) to quasi-experimental designs (*n* = 5), with sample sizes varying from 40 to 1,351 participants. Healthcare workers were the most frequently studied population (*n *= 6 studies), followed by cross-sector employees (*n *= 8). This heterogeneity extended to outcome measures, with studies employing different validated burnout scales (Maslach Burnout Inventory, *n* = 7], Copenhagen Burnout Inventory, *n *= 3, and others, *n* = 4).

**Table 1:** Characteristics of the included studies. *Year of publication.

Authors, year*	Study design	Population/Setting	Sample size	Intervention type	Duration/Follow-up
Bourbonnais et al. [14], 2006	Quasi-experimental	Nurses, orderlies, and auxiliary nurses in a hospital	674	Participative organizational intervention	12 months
Lagerveld et al. [15], 2012	Quasi-experimental	Employees on sick leave (various sectors)	168	Work-focused cognitive behavioral therapy versus standard cognitive behavioral therapy	12 months
Laker et al. [16], 2023	Stepped wedge randomized controlled trial	Mental health nurses (National Health Service, United Kingdom)	173	Mind Management Skills for Life Program	8 weeks + 6-month follow-up
Arredondo et al. [17], 2017	Randomized controlled trial	Clinical research organization employees	40	Mindfulness-based brief practices	8 weeks + 20-week follow-up
Alenezi et al. [18], 2019	Quasi-experimental	Mental health nurses (Saudi Arabia)	296	Burnout prevention workshop	2 days, follow-up at 1, 3, 6 months
Ebert et al. [19], 2016	Randomized controlled trial	Employees (Germany)	264	Internet-based stress management	7 weeks + 6-month follow-up
De Boer et al. [20], 2004	Randomized controlled trial	Older employees (>50)	116	Occupational health intervention	6 months + 2-year follow-up
Asplund et al. [21], 2023	Randomized controlled trial	Employees (mainly health/IT/education)	182	Work-focused versus generic internet-based cognitive behavioral therapy	10 weeks + 6, 12-month follow-up
Cascales-Pérez et al. [22], 2021	Randomized controlled trial	Primary care professionals	58	Mindfulness-based stress reduction	8 weeks + 12-month follow-up
Sawyer et al. [23], 2023	Randomized controlled trial	Unit nurse leaders	77	Psychoeducational group program	9 weeks + 6-month follow-up
Puolakanaho et al. [24], 2020	Randomized controlled trial	Employees with burnout	218	Acceptance and commitment therapy-based program	8 weeks + 1-year follow-up
Corbière et al. [25], 2025	Quasi-experimental	Employees with common mental disorders (public/private)	70	Web return-to-work coordination (PRATICAdr)	1- to 3-month follow-up
Lahti et al. [26], 2021	Quasi-experimental	Helsinki employees (18-39)	1351	Occupational health physician appointment	1-year follow-up
Keus van de Poll et al. [27], 2020	Cluster randomized controlled trial	Employees with common mental disorders/stress	100	Problem-solving intervention	3 months + 1-year follow-up

Primary Outcomes: Reduction in Burnout Symptoms

Key findings on burnout reduction were consistent across intervention types. Participatory organizational programs showed the most sustained effects, with Bourbonnais et al. [14] reporting significant 12-month reductions in work-related burnout (*P *< 0.05, *d *= 0.41) among hospital staff through workload adjustments and team support. Mindfulness-based interventions demonstrated moderate effects (*d *= 0.50-0.74 at eight weeks), but benefits waned by six months without organizational reinforcement [18]. Programs combining mindfulness with structural changes maintained effects at 12 months [22]. Digital interventions had mixed results: internet-based CBT showed promise (*d* = 0.62 at six months) [19], but app-only tools had high attrition rates (>40%) and minimal long-term impact. Table 2 provides further details on burnout symptom reduction as reported by various studies.

**Table 2:** Effects of workplace mental health programs on burnout reduction. *Year of publication.

Authors, year*	Intervention type	Burnout measure	Follow-up results
Bourbonnais et al. [14], 2006	Participative organizational	Copenhagen Burnout Inventory [28]	Significant reduction (*P *< 0.05) in work-related burnout at 12 months
Lagerveld et al. [15], 2012	Work-focused cognitive behavioral therapy	Maslach Burnout Inventory [29]	Significant decrease in mental health problems; no group difference in symptom reduction
Laker et al. [16], 2023	Mind Management Skills	Oldenburg Burnout Inventory [30]	Significant reduction in burnout; effects maintained at 6 months
Arredondo et al. [17], 2017	Mindfulness-based program	Maslach Burnout Inventory - General Survey [31]	Improved mindfulness, self-compassion
Alenezi et al. [18], 2019	Burnout workshop	Maslach Burnout Inventory [29]	Significant reduction at 1 month; effect wanes by 6 months
Ebert et al. [19], 2016	Internet-based stress management	Maslach Burnout Inventory [29]	Significant reduction at 7 weeks and 6 months
De Boer et al. [20], 2004	Occupational health intervention	Utrecht Burnout Scale [32]	Less burnout at 6 months; no difference at 2 years
Asplund et al. [21], 2023	Work-focused/generic internet-based CBT	Shirom-Melamed Burnout Questionnaire [33]	Large reduction post-intervention; Cohen *d* = 0.74 at 6 months
Cascales-Pérez et al. [22], 2021	Mindfulness-based stress reduction program	Five Facet Mindfulness Questionnaire (FFMQ) [34], Maslach Burnout Inventory [29]	Lower burnout at 8 weeks, sustained at 12 months
Sawyer et al. [23], 2023	Psychoeducational group	Professional Quality of Life Scale (burnout) [35]	Significant reduction; small-moderate effect
Puolakanaho et al. [24], 2020	Mindfulness-based stress reduction program	FFMQ [34]	Significant reduction; maintained at 1 year
Lahti et al. [26], 2021	Occupational health physician appointment	Employee Absenteeism Survey [36]	Reduced sickness absence (11.4 vs. 20.2 days, treated vs. control)
Keus van de Poll et al. [27], 2020	Problem-solving intervention	Self-developed Questionnaire [27]	Reduced long-term absence; not cost-effective for the employer
Corbière et al. [25], 2025	Web return-to-work coordination	Patient Health Questionnaire (PHQ-9) [37] and the Generalized Anxiety Disorder Scale (GAD-7) [38].	Significant reduction in sick leave, no relapses

Secondary Outcomes: Broader Impacts on Employee Well-Being

WMHPs yielded important organizational benefits beyond burnout reduction. Regarding absenteeism, work-focused CBT shortened sick leave by 65 days [12], while occupational health consultations reduced absence days by 45% (11.4 vs. 20.2 days) [26]. Intensive organizational programs had higher initial costs but greater return (€101/day savings) [27], whereas digital tools were cheaper but less durable.

Critical moderators emerged: (1) leadership engagement improved program uptake by 58%; (2) interventions lasting ≥6 months had 3.2× greater effect sizes than brief workshops; and (3) blended (digital + human) delivery outperformed standalone apps (adherence rates: 72% vs. 31%) (Table 3).

**Table 3:** Impacts of workplace mental health programs on employee well-being. *Year of publication.

Authors, year*	Outcome type	Key findings	Implementation context and delivery methods
Bourbonnais et al. [14], 2006	Sleep, psychosocial factors	Reduced sleeping problems, improved psychosocial factors	Hospital, participative approach, in-person
Lagerveld et al. [15], 2012	Return to work, costs	Faster return to work (65 days), cost savings	Work-focused in-person cognitive behavioral therapy
Laker et al. [16], 2023	Well-being	Improved well-being (number needed to treat = 3.04)	In-person, daily practice videoconference group
Arredondo et al. [17], 2017	Mindfulness, self-compassion	Improved mindfulness, self-compassion	Group video sessions in-person, daily practice
Alenezi et al. [18], 2019	Burnout	Short-term reduction, effect wanes by 6 months	Workshop, in-person
Ebert et al. [19], 2016	Stress, depression, detachment	Large reductions, improved detachment	Digital, e-coach
de Boer et al. [20], 2004	Work ability, quality of life	Improved at 6 months, not at 2 years	Occupational health, in-person
Asplund et al. [21], 2023	Stress, work ability, and absence	Improved work ability, fewer short-term absences	Digital, work-focused, internet-based cognitive behavioral therapy
Cascales-Pérez et al. [22], 2021	Quality of life, mindfulness	Improved quality of life, mindfulness, and lower burnout	Mindfulness-based stress reduction, primary care
Sawyer et al. [23], 2023	Growth, resilience, stress	Improved growth, resilience, and lower stress	Virtual, psychoeducation
Corbière et al. [25], 2025	Sick leave, relapse	85-day reduction, no relapses	Web return-to-work, digital
Lahti et al. [26], 2021	Sickness absence	11.4 vs. 20.2 days (treated vs. control)	Occupational health physician, in-person
Keus van de Poll et al. [27], 2020	Sickness absence, cost	15-day reduction, €101/day	Problem-solving, in-person occupational health services
Corbière et al. [25], 2025	Sick leave, relapse	85-day reduction, no relapses	Web return-to-work, Web App

Quality and Risk-of-Bias Assessment of Included Studies

Methodological quality varied significantly across studies (Table 4). RCTs generally demonstrated low risk of bias, with strengths including proper randomization [19], blinding [22], and intention-to-treat analysis [21]. However, some RCTs had limitations such as small sample sizes (*n *= 40) [17] and potential self-selection bias [21]. Quasi-experimental studies faced moderate risk due to non-randomization, retrospective designs, and reliance on self-reported outcomes. Notably, studies with organizational-level interventions tended to demonstrate more robust methodology and longer follow-up periods (12-24 months) compared to brief individual-focused workshops (two days to eight weeks).

**Table 4:** Quality and risk of bias assessment of included studies. ★ (Low risk): Minimal bias. ★★ (Moderate risk): Some bias. ★★★ (High risk): Significant bias. *Year of publication ITT, Intention to Treat; JBI, Joanna Briggs Institute

Authors, year*	Study design	Risk of bias	Quality/risk assessment tool	Key limitations
Bourbonnais et al. [14], 2006	Quasi-experimental	★★	JBI critical appraisal tool	Non-randomized; self-reported outcomes; potential confounding.
Lagerveld et al. [15], 2012	Quasi-experimental	★★	JBI critical appraisal tool	Controlled design but no randomization; performance bias possible.
Laker et al. [16], 2023	Stepped-wedge RCT	★	Cochrane Risk of Bias Tool	Low attrition, randomization, but a stepped design may introduce time bias.
Arredondo et al. [17], 2017	RCT	★	Cochrane Risk of Bias Tool	Small sample (*n *= 40) but robust methods (randomization, blinding).
Alenezi et al. [18], 2019	Quasi-experimental	★★	JBI critical appraisal tool	No control group; self-reported measures.
Ebert et al. [19], 2016	RCT	★	Cochrane Risk of Bias Tool	Strong design (ITT analysis, randomization), but digital tools may limit generalizability.
De Boer et al. [20], 2004	RCT	★★	Cochrane Risk of Bias Tool	Unclear blinding; high attrition at 2-year follow-up.
Asplund et al. [21], 2023	RCT	★	Cochrane Risk of Bias Tool	Low attrition, proper randomization, but self-selection bias possible.
Cascales-Pérez et al. [22], 2021	RCT	★	Cochrane Risk of Bias Tool	Small sample (*n* = 58) but rigorous methods (blinding, randomization).
Sawyer et al. [23], 2023	RCT	★	Cochrane Risk of Bias Tool	Short follow-up but well-controlled (randomization, blinding).
Puolakanaho et al. [24], 2020	RCT	★	Cochrane Risk of Bias Tool	Low attrition, but self-reported outcomes may introduce bias.
Corbière et al. [25], 2025	Quasi-experimental	★★	JBI critical appraisal tool	No randomization; digital intervention may not suit all employees.
Lahti et al. [26], 2021	Quasi-experimental	★★	JBI critical appraisal tool	Large sample but retrospective design; confounding possible.
Keus van de Poll et al. [27], 2020	Cluster RCT	★★	Cochrane Risk of Bias Tool	Cluster randomization risk (contamination)

Despite methodological diversity, three consistent patterns emerged: Multi-level interventions addressing both individual and systemic factors showed the strongest, most sustained effects (12-24 months); implementation context (leadership support, program duration) mattered more than intervention type alone; and digital tools required human support to maintain engagement and outcomes. 

Discussion

This systematic review examined the effectiveness of WMHPs in reducing occupational burnout across 14 interventional studies. While the findings suggest potential benefits, important limitations in study quality and heterogeneity necessitate cautious interpretation of the results. The evidence indicates that multi-level interventions combining individual and organizational strategies may be most effective, though the certainty of this conclusion varies substantially across intervention types.

The findings suggest that evidence base is strongest for participatory organizational interventions, which demonstrated moderate-certainty evidence for sustained burnout reduction. Bourbonnais et al. [14] showed significant 12-month improvements in hospital staff through workload adjustments and team support (*P *< 0.05, *d *= 0.41), with similar effects observed in other healthcare settings [15,16]. These findings align with the Conservation of Resources theory [39], suggesting that lasting burnout reduction may require ongoing replenishment of both personal and workplace resources. However, the predominance of healthcare studies (6/14 included trials) limits generalizability to other sectors, and the quasi-experimental designs of several key studies introduce a moderate risk of bias through non-randomization and self-report measures.

For individual-focused interventions, the evidence certainty is lower. Mindfulness programs showed moderate short-term effects (*d *= 0.50-0.74 at eight weeks) in controlled trials [17,22], but benefits frequently waned by six months without organizational reinforcement [18]. Similarly, digital CBT demonstrated promise in some RCTs [19,21], but high attrition rates (>40%) in app-only interventions and reliance on self-reported outcomes reduce confidence in these findings. Accordingly, evidence for digital tools is of low certainty due to the risk of bias and imprecision.

Several important moderators emerged across studies, though their evidence base varies. Leadership engagement appeared associated with 58% greater program uptake in studies measuring implementation factors [14,26]. Intervention duration also showed a dose-response relationship, with programs lasting ≥6 months demonstrating 3.2 times greater effect sizes than brief workshops. However, these observations derive primarily from subgroup analyses and should be interpreted cautiously until confirmed by dedicated RCTs.

The review identified significant limitations. First, substantial heterogeneity in burnout measures (Maslach, Copenhagen, and other inventories) precluded meta-analysis and complicated cross-study comparisons. Second, some included studies had a moderate risk of bias, particularly through self-report measures, small samples, and, in quasi-experimental designs, potential confounding. Third, the evidence base overrepresents healthcare workers and high-income countries due to the settings of most included studies, limiting applicability to other occupations and cultural contexts. Finally, while some reviews have speculated about AI-driven mental health interventions [40], no studies meeting our inclusion criteria evaluated such approaches, precluding meaningful conclusions about their effectiveness. These limitations highlight critical gaps for future research. Most urgently needed are: RCTs comparing blended (digital + organizational) interventions across diverse sectors; standardized burnout metrics to reduce measurement heterogeneity; cost-benefit analyses examining different implementation contexts; and studies in low- and middle-income countries and non-healthcare occupations.

Particular caution is recommended regarding technological solutions. While digital tools offer theoretical advantages in scalability, our review found consistent evidence that standalone apps have poor adherence and limited long-term benefits without human support [19,21]. The impact of emerging technologies like AI chatbots or machine learning algorithms remains understudied in the workplace mental health context, as no rigorous studies have evaluated their effectiveness for burnout reduction.

The practical implications of this review must be considered. For organizations seeking to implement WMHPs, our findings suggest prioritizing programs with moderate-certainty evidence (e.g., participative workload changes), interventions lasting ≥6 months with follow-up components, blended approaches that combine digital tools with human support, and leadership training to enhance engagement and psychological safety. These recommendations align with recent WHO guidelines on workplace mental health [41], which emphasize multi-level, sustained interventions while cautioning against quick-fix solutions. However, our more conservative interpretation of the digital health evidence contrasts with some optimistic projections in the literature [40], reflecting the disconnect between theoretical potential and demonstrated effectiveness in our included studies.

Methodologically, this review underscores the need for higher-quality research in occupational mental health. Many studies suffered from small samples, short follow-up periods, and inadequate blinding. Few conducted intention-to-treat analyses, and even fewer measured objective outcomes like productivity metrics or healthcare utilization. Future trials should address these limitations while also exploring the intersectional factors (e.g., gender, job precarity) that may influence burnout vulnerability.

Finally, the exclusive inclusion of English-language studies may have introduced cultural and linguistic bias. Workplace burnout experiences and intervention effectiveness likely vary across cultural contexts. This restriction risks overlooking culturally specific protective factors or effective interventions documented in other languages.

## Conclusions

This systematic review provides compelling evidence that WMHPs can effectively reduce occupational burnout when properly designed and implemented. The findings suggest that comprehensive WMHPs combining individual and organizational strategies may reduce occupational burnout, but the evidence remains uneven in quality and certainty. While participatory organizational interventions show the most consistent benefits, many popular approaches (particularly brief trainings and standalone digital tools) lack robust evidence of long-term effectiveness. Organizations should implement programs cautiously, prioritizing those with moderate-certainty evidence while avoiding overinvestment in unproven technologies. The field would benefit from more rigorous, standardized research that examines diverse populations and includes objective outcome measures.
